# Abdominal obesity is an independent predictor of serum 25-hydroxyvitamin D deficiency in adults with cerebral palsy

**DOI:** 10.1186/1743-7075-11-22

**Published:** 2014-05-19

**Authors:** Mark D Peterson, Heidi J Haapala, Ashish Chaddha, Edward A Hurvitz

**Affiliations:** 1 Department of Physical Medicine and Rehabilitation, University of Michigan Health Systems, 325 E. Eisenhower Parkway, Suite 300, Ann Arbor, Michigan 48108, USA

**Keywords:** Cerebral palsy, 25-hydroxyvitamin D, Visceral adiposity, Motor function

## Abstract

**Background:**

Individuals with cerebral palsy (CP) are at risk for nutritional insufficiency. The purpose of the study was to examine the vitamin D status of adults with CP, and to evaluate the association between vitamin D and functional level, age, race, and anthropometric indicators of adiposity.

**Methods:**

Serum vitamin D levels, BMI, waist circumference (WC), and functional level (measured by Gross Motor Function Classification System (GMFCS)) were examined in 112 adults with CP. Vitamin D status was assessed by serum 25-hydroxyvitamin D level (25(OH)D). The influence of motor impairment and adiposity on 25(OH)D were assessed using general linear modeling and logistic regression, with age, sex, race, and season as covariates.

**Results:**

Mean vitamin D was 28.1 ± 16.0 ng/ml. Only 45% of subjects had optimal levels of 25(OH)D, 21% were insufficient and 34% were deficient. Overweight or obesity was prevalent (52%), as was abdominal obesity in men (23.5% at 102 cm cutoff) and women (31.1% at 88 cm cutoff). There was a robust association between the indicator of visceral adiposity (WC) and 25(OH)D level (p <0.001), even after controlling for age, sex, race, season, and GMFCS. According to sex-specific WC cutoffs, the odds of being deficient in vitamin D increase by a factor of 3.5 (95% CI 1.12-11.0) for abdominal obesity. GMFCS was not associated with 25(OH)D.

**Conclusions:**

Adults with CP are at risk for low vitamin D levels and overweight/obesity. Waist circumference is a strong independent predictor for low vitamin D levels.

## Background

Vitamin D plays an important role in a wide range of organ functions, and is suggested to contribute significantly to cardiometabolic and musculoskeletal health. Vitamin D deficiency is now considered to be an epidemic, with recent estimates as high as 64% of the general healthcare population [1]. At present there is substantial debate regarding the optimal level of serum 25-hydroxyvitamin D (25(OH)D) as well as the efficacy and dosing strategy of supplemental vitamin D; however, most reports agree that serum levels less than 20 ng/mL (i.e. 50 nmol/L) may be insufficient for optimal bone health [2]. Historically considered an independent predictor of osteoporosis [3] and muscle function [4,5], emerging evidence has also linked vitamin D deficiency with a host of obesity-related comorbid conditions such as metabolic syndrome, coronary heart disease, hyperlipidemia, and diabetes [1,6,7], as well as early mortality [8]. Indeed, there is now a well-established reciprocal association between adiposity and vitamin D, which may be caused by a decreased bioavailability of and deposition in adipose tissue compartments [9-11] (particularly in the visceral depot [12,13]).

The reported incidence of vitamin D deficiency is population specific, and contingent upon various demographic factors such as age, sex, race and geographical location (i.e., distance from the equator, and/or locations wherein foods are not fortified with vitamin D) [2]. These general characteristics notwithstanding, select sub-populations such as individuals with congenital or chronic neuromuscular disorders (e.g., cerebral palsy (CP)) may be at heightened risk for hypovitaminosis D-induced osteopenia and impaired bone mass accrual [14], as well as secondary health complications associated with or exaggerated by the combination of insufficient vitamin D [15], chronic sedentary behavior, and obesity [16]. Although at present the role of vitamin D on secondary health complications in CP is unknown, it is certainly conceivable that degree of motor impairment and abdominal adiposity may serve as significant predictors for vitamin D status.

However, most clinicians who treat patients with CP do not check vitamin D levels on a consistent basis. Considering the inherent feeding difficulties associated with esophageal reflux, swallowing, and gastric disorders, many CP patients have poor overall nutrition and insufficient calcium and vitamin D intake. Although a large body of research has confirmed a significant association between nutritional insufficiency, growth, and diminished musculoskeletal health in CP, patients are also routinely prescribed anticonvulsant medications that interfere with calcium and vitamin D absorption, and that further contribute to low bone mineral density [17]. Moreover, and in combination with inadequate nutrient intake/absorption, chronic pain and fatigue in CP represent two significant barriers for participation in physical activity and load bearing exercise [18], and thus dramatically increase the likelihood of being sedentary and housebound with little exposure to sunlight.

The purpose of this study was to examine vitamin D status among adults with CP, and to determine the associations with gross motor function and several anthropometric indicators of adiposity, after adjustment for known covariates such as age, season, and race. Since degree of motor impairment is a strong indicator of nutritional insufficiency and chronic sedentary behavior, we hypothesized that vitamin D levels would be independently associated with more severe motor impairment. We also hypothesized that both BMI and waist circumference would be independent predictors of lower serum vitamin D. Certainly, understanding the independent contributions of motor impairment, as well as a modifiable predictor such as adiposity, on vitamin D status, may better define risk for latent complications which may exacerbate the CP condition.

## Methods

### Study design

Patient records were obtained through the University of Michigan Adult Cerebral Palsy Clinic, Department of Physical Medicine and Rehabilitation. This retrospective study included a synthesis of relevant patient descriptive data, as well as bivariate and regression analyses between various anthropometric characteristics (i.e., body mass, height, BMI, waist circumference and hip circumference, and waist-to-hip ratios), functional status, and serum vitamin D.

### Patient population

Male and female participants were 18 years and older, admitted to University of Michigan adult CP clinic. Patients in the sample represented a variety of diagnostic categories for CP, according to the Gross Motor Function Classification System (GMFCS). The initial sample consisted of 130 patients’ clinic records that were eligible for inclusion in the analysis. We excluded records that did not include sufficient data pertaining to vitamin D status and/or general intake patient characteristics such as anthropometric measures. The final sample included 112 patient records (52 males, 60 females; age = 34 ± 13.4 years). Race/ethnicity distribution was primarily Non-Hispanic white (~90%), as compared to African American (8%) and Mexican American or other Hispanic (~2%). Each subject signed an informed consent document and all procedures were approved by the University of Michigan Medical institutional review board for research with human subjects.

### Measures

#### ***Gross motor function classification system (GMFCS)***

The Gross Motor Functional Classification System (GMFCS) assesses activity limitations for gross motor function with a five level ordinal grading scale. The GMFCS in the adult population has been shown to be reliable (interclass correlation coefficient (ICC) = 0.93) and have excellent interrater reliability (quadratic kappa value of 0.978) [19]. All patients were examined by the same physician investigator to classify GMFCS level-which was available upon chart review. The individuals were also classified into two severity categories: GMFCS levels I-III and GMFCS levels IV-V, as previously documented [20].

#### ***Anthropometric measures***

As a part of normal medical intake, each patient was tested for body mass (kg), height (cm), and body mass index (BMI-kilograms per squared meters [(kg∙m^-2^]). For patients who had difficulty standing erect, supine recumbent length had been measured and was used instead to calculate height. Waist circumference (WC) and hip circumference (HC) were measured using a standard Gulick tape, and evaluated to the nearest 0.1 cm. For WC, a horizontal measure was taken at the narrowest part of the torso (i.e. below xiphoid process and above the umbilicus). Standard cutpoints for abdominal obesity in men (>102 cm) and women (>88 cm) were used, as outlined by the ATP III report. For HC, horizontal measures were taken at the site of maximal circumference. Waist-to-hip ratio (WHR) was also calculated.

#### ***Vitamin D status and parathyroid hormone (PTH)***

Vitamin D levels were drawn as part of the patients’ clinical evaluation. Blood samples were drawn by venipuncture after a 12-h fast, and assayed for serum 25(OH)D at the University of Michigan Clinical Chemistry Laboratories, in Department of Pathology. Determinations of 25(OH)D were made in serum by a chemiluminescence immunoassay (CLIA) with intra- and interassays variabilities of 4.7% and 7.1% respectively. Vitamin D status was categorized as optimal (≥30 ng/ml), insufficient (21–29 ng/ml), and deficient (<20 ng/ml), based on the recommended cut-points from the Endocrine Society [21], as well as recommendations from the Institute of Medicine (IOM) [22]. Moreover, due to the known seasonal variation of serum 25(OH)D [23], we statistically adjusted for the corresponding season of blood draw. Intact serum PTH was analyzed using a chemiluminescence assay. The reference range is 10–65 pg/mL, and the interassay coefficient of variation was <4.3% at 36 pg/ml.

### Statistical analysis

All statistical analyses were performed using SAS software version 9.3 (SAS Institute, Cary, NC). Descriptive characteristics were stratified by GMFCS (i.e. GMFCS I-III and IV-V). Differences between GMFCS levels were determined for general demographic and anthropometric characteristics as well as vitamin D (25(OH)D) by an independent-sample t-test, and difference in proportions for sex by Chi-square test. Pearson product–moment correlations were used to examine selected bivariate correlations. A minimum criterion alpha level of p ≤ 0.05 was used to determine statistical significance. Data are reported as means and standard deviations (SD). Linear regression was used to determine differences in vitamin D (25(OH)D) between GMFCS levels, seasons, and races. This process was completed over several steps that required creating dummy codes for each season, race, and GMFCS level (i.e., category). Thereafter, we examined vitamin D status for differing categories by systematically removing one category at a time (and thus creating an interchangeable reference). Using this procedure, the β coefficient for the intercept represented the mean of the removed category, and the regression provided coefficients for each subsequent category, a standard error, a t-value, and a p-value. Partial correlation and multiple linear regression were also used to evaluate the associations between vitamin D (25(OH)D), GMFCS, and various indicators of adiposity, after controlling for multiple potential moderating factors. In addition to the predictors, the following standard covariates were included in the original model: age, sex, race, and season. A logistic model was also fitted (i.e. using the PROC LOGISTIC procedure) with explanatory variables to determine a best fit model for categorical vitamin D deficiency. Dummy coding was applied to the categorical variables (e.g., GMFCS I-III = 0, GMFCS IV-V = 1). Normality of the residuals was tested using a Shapiro-Wilks test and homogeneity of the variance of the residuals was tested with standard regression diagnostics. Multicollinearity was tested using a variance inflation factor (VIF). Tests revealed no issues of collinearity for any model. Initial model testing revealed that sex was not a primary predictor of vitamin D status (β = 0.09; p = 0.36), and therefore we chose not to stratify by sex, but rather to include sex as a categorical variable in the final models.

## Results

### Subject characteristics

Characteristics of the study population are presented in Table 1. Of the 112 patient records that were included in the analysis, 53.6% were female (n = 60). GMFCS level ranged from I-V, with the following breakdown (GMFCS I: *n* = 9 [8.0%]; GMFCS II: *n* = 20 [17.9%]; GMFCS III: *n* = 29 [25.9%]; GMFCS IV: *n* = 29 [25.9%]; and GMFCS V: *n* = 25 [22.3%]. Based on traditional cut points, approximately 52% of patients were overweight or obese (i.e., BMI ≥ 25 kg∙m^-2^), and of those, 23.5% were classified as obese (i.e., BMI ≥ 30 kg∙m^-2^). Adults with GMFCS IV-V were generally smaller in stature than GMFCS I-III.

**Table 1:** Descriptive characteristics of all adults with cerebral palsy, and differences between Gross Motor Function Classification System (GMFCS) I-III and IV-V

	**Total Sample**	**GMFCS I-III**	**GMFCS IV-V**	**p-value**
N	112	58	54	
Female. O	53.6	51.4	52.0	0.805
Age On)	34.00 (13.41)	32.95 (13.63)	36.10 (18.01)	0.224
Race/Ethnicity, %				
Non-Hispanic White	90.4	87.9	92.3	0.45
African American	7.8	10.3	5.8	0.39
Mexican American or Other Hispanic	1.7	1.7	1.9	0.94
Body Mass (kg)	61.73 (25.09	72.35 (22.51)	51.02 4.73)	<0.001*
Recumbent Length (cm)	159.21 (13.35)	161.65 (9.90)	155.68 (16.27)	0.043*
Body Mass Index (kg∙m^-2^)	25.86 (7.59)	28.72 (7.41)	22.79 (634)	<0.001*
Obesity (BMI≥30), %	23.5	32.1	14.00	0.04*
Waist Circumference (cm)	85.53 (20.48)	92.80 (17.47)	79.20 (22.14)	0.003*
Hip Circumference (cm)	93.75 (17.23)	101.19 (1 3.10)	85.06 (18.57)	<0.001*
Waist-to-Hip Ratio	0.90 (0.09)	0.90 (0.10)	0.90 (0.08)	0.74
25-hydroxyvitanin D (25(OH)D) (ng/mL)	30.17 (15.02)	27.53 (11.68)	31.71 (11.40)	0.143
Parathvroid hormone (PTH). (pg/mI)	49.8 (26.65)	47.96 (25.10)	52.63 (28.86)	0.418

*Significant difference between GMFCS I-III and IV-V (p <0.05).

### Vitamin D level

**Figure 1:**
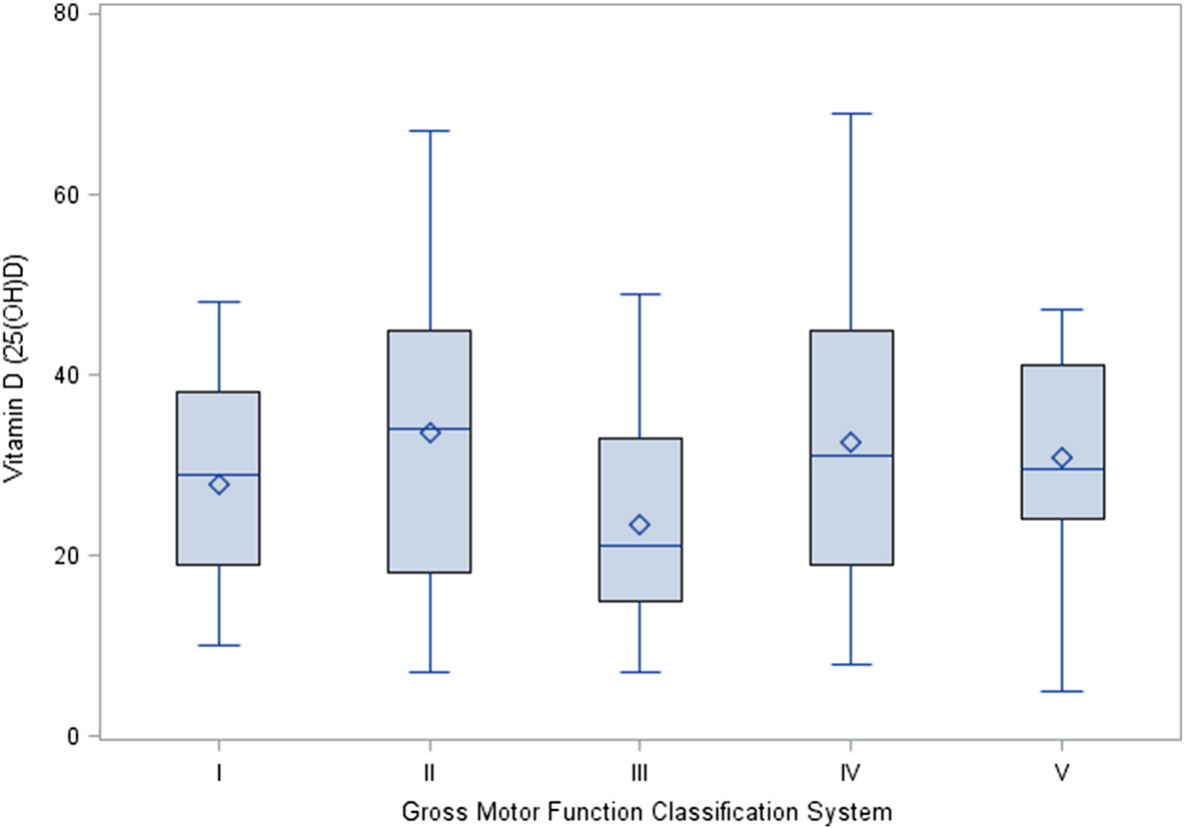
**Comparison of mean and individual 25-hydroxyvitamin D concentrations across levels of Gross Motor Function Classification System (GMFCS).** There were no significant differences between the levels as assessed by linear regression.

The range for vitamin D level was from 5 to 64 ng/mL (Table 1), and correlated to both parathyroid hormone (PTH) (r = -0.24; p = 0.023) and WC (r = -0.40; p = 0.002). Vitamin D was not correlated to age, BMI, WHR, or HC. 52 subjects (45.2%) were considered to have optimal levels of vitamin D (>30 ng/mL); whereas 20.9% had insufficient levels (21–29 ng/mL); and 33.9% were deficient (<20 ng/mL). There were no differences in vitamin D status across levels of GMFCS (p = 0.092) (Figure 1). The largest seasonal difference in vitamin D was between winter and summer (β = -4.14 ng/mL), but was not significant (p = 0.3067). African Americans had lower 25(OH)D (β = -11.45; *p* = 0.02) than Non-Hispanic white subjects.

**Figure 2:**
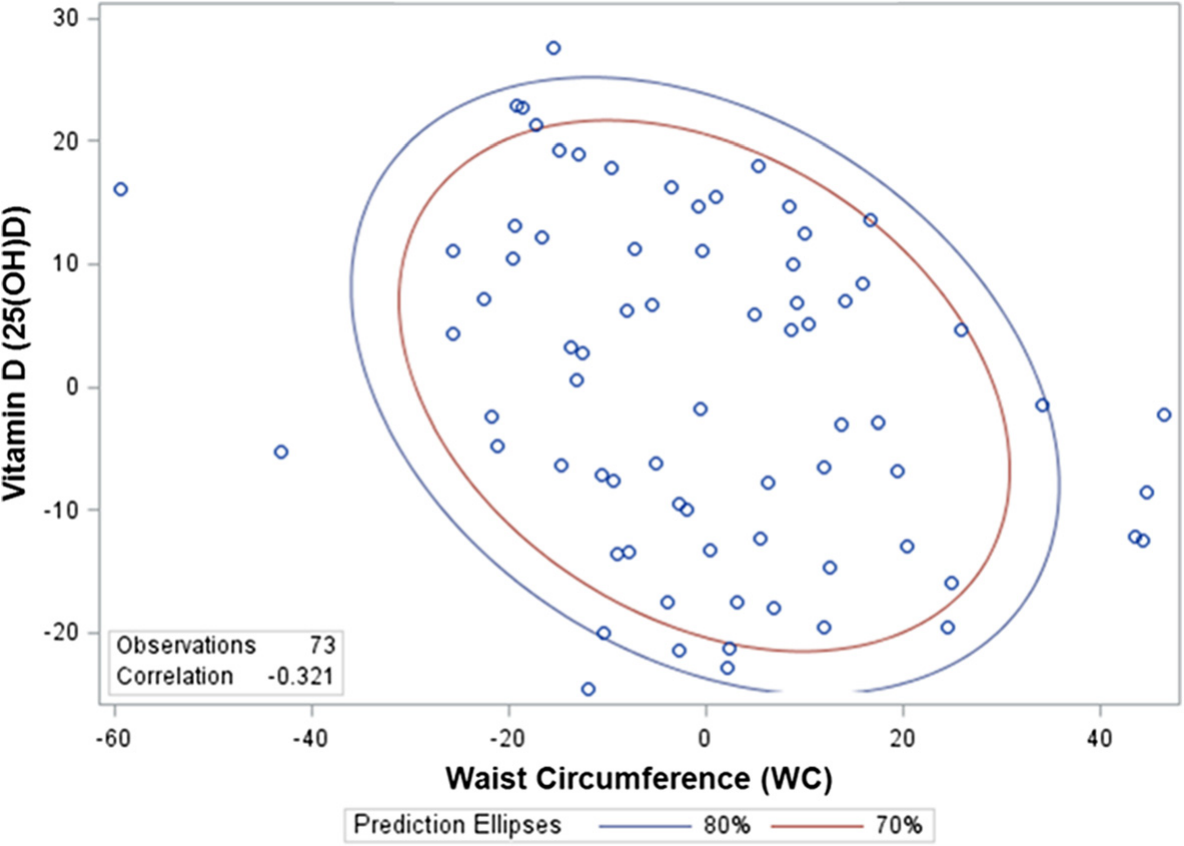
Partial residual scatter plot for the variables waist circumference (WC) and 25-hydroxyvitamin D after controlling for the effect of variables age, sex, and GMFCS (with 70% and 80% prediction ellipses).

Multiple linear regression examining predictors of vitamin D (25(OH)D) status identified WC (β = -0.27; *p* < 0.001) (see: Figure 2 for Partial residual scatter plot) as significant, controlling for age, sex, race, season, and GMFCS (Table 2). These findings reveal that greater central adiposity is associated with lower vitamin D status, independent of motor impairment.

**Table 2:** General linear model to determine the independent association of functional impairment (GMFCS) and waist circumference (WC) on vitamin D status, adjusting for age, sex, GMFCS, Race and Season

	**Model predictor(s)**	**β**	**SE**	**t**	**Pr > |t|**	**Adjusted R**^**2**^
25 hydroxyvitamin D (25(OH)D)						0.19
	Intercept	55.56	l0.1	5.46	<0.001	
	Age	0.0	0.13	0.59	0.55	
	Sex	-0.66	3.23	-0.20	0.84	
	WC	-0.27	0.08	-3.35	<0.001	
	GMFCS	-0.47	1.4	-0.32	0.74	
	Race/Ethnicity	-11.74	4.02	-2.91	0.01	
	Season	-1.56	1.52	1.02	0.31	

Logistic regression was conducted to identify the odds of being deficient at the level published by the Endocrine Society and IOM report [21,22], and revealed that for every 10 cm increase in WC, the odds of being deficient in vitamin D increase by a factor of 1.5 (95% CI: 1.062 - 1.974). According to sex-specific WC cutoffs, the odds of being deficient increased by a factor of 3.5 (95% CI 1.12-11.0) for abdominal obesity.

Although a dichotomous cutoff for GMFCS was used to create the motor impairment groups, 25(OH)D concentrations varied significantly. Thus, as an additional way to present the association between vitamin D and indicators of adiposity, we stratified subjects on the basis of 25(OH)D quartiles (Table 3). Subjects with higher vitamin D levels, across quartiles, had incrementally lower WC measures.

**Table 3:** Comparison of variables in quartiles of 25(OH)D concentration

	**Quartile 1**	**Quartile 2**	**Quartile 3**	**Quartile 4**	**p value**
	**(n = 27)**	**(n = 32)**	**(n = 26)**	**(n = 30)**	
25-hydroxnitamin D (25(OH)D) (ng/mL)	11.13 (3.51)	22.34 (3.85)	34.50 (3.62)	49.37 (8.10)	NA
Age (yrs)	33.67 (15.6)	35.98 (13.87)	36.06 (12.56)	30.35 (1 L20)	0.317
Female (%)	44.00	*26.67*	18.30	36.67	0.082
GMFCS I-III (%)	29.31	25.86	25.86	18.97	0.201
GMFCS IV-V (%)	19.23	30.77	21.15	28.85	0.716
Body Mass (kg)	68.31 (28.3)	64.86 (21.37)	66.94 (23.28)	47.62 (22.79	0.006
Recumbent Length (cm)	158.35 (12.92)	159.14 (8.29)	161.99 (16.83)	157.3 (15.36)	0.684
Body Mass Index (kg∙m^-2^)	28.90 (9.9 M)	25.22 (7.23)	26.51 (6.18)	22.81 (5.47)	0.052
Waist Circumference (cm)	93.75 (19.06)	89.56 (24.73)	85.68 (17.01)	73.95 (15.60)	0.012
Hip Circumference (cm)	103.91 (17.34)	92.5 (13.62)	94.08 (12.77)	84.41 (19.78)	0.001
Parathyroid hormone (PTH). (pg/ml)	59.32 (32.87)	55.32 (26.83)	4 L89 (21.49)	40.84 (18.14)	0.036

p-value are presented to represent significant trend testing across quartiles.

## Discussion

The primary finding of this investigation is that adults with CP are at risk for insufficient and deficient serum vitamin D; however, vulnerability was not associated with the severity of motor impairment. It is well known that severely impaired individuals with CP experience significant feeding issues and are at greater risk for malnutrition, which are in-turn thought to be the primary drivers of musculoskeletal fragility and altered growth trajectories in this population. Interestingly, we found that individuals classified in the highest severity category (i.e., GMFCS V) had a similar serum vitamin D (25(OH)D) status (30.8 ng/mL) as individuals in the least impaired category (i.e., GMFCS I) (27.9 ng/mL). This does not imply that severity of motor impairment is unrelated to nutrition status, growth, and/or bone health in CP; however, it *does* highlight an independent factor that may pose an even greater risk for vitamin D insufficiency. The overall prevalence of vitamin D insufficiency or deficiency was approximately 56%, which is similar to the typically-developed adult population [21,24]. Interestingly, unlike the general population [25], BMI was not associated with serum 25(OH)D in this study.

Rather, abdominal obesity was the strongest predictor for serum vitamin D in this heterogeneous sample of adults with CP, even after adjusting for age, sex, race, season, and level of motor function. Indeed, there is a well-established link between vitamin D deficiency, abdominal obesity and cardiometabolic risk [12,26], and yet most studies do not account for at-risk populations with severe motor disabilities. Moreover, although several studies have identified a general increased prevalence of obesity among children with CP [27,28], the influence of obesity on secondary health complications has yet to be studied through the lifespan. It is of course logical to presume that the primary neurological insult associated with CP is the underlying cause for impaired growth and risk for musculoskeletal deterioration, but it is also likely that excessive central adiposity may lead to exaggerated risk for a non-CP, comorbid sequela as children with CP transition into adulthood. The current findings clearly support the need for greater clinical attention to assessments of serum vitamin D status in all patients with CP. Particular attention should also be given to individuals with CP who are African American, as vitamin D was significantly lower as compared to Non-Hispanic white individuals.

Moreover, children and adults with CP have significantly diminished lean body mass [29] and greater intermuscular adipose tissue [30], which collectively highlights the implications of skeletal muscle deterioration. Thus, even normal BMIs in this population may disguise excess body fat (i.e., “obesity misclassification” [31]), and risk for cardiovascular and metabolic dysregulation. Based on standard BMI cutoffs, our data revealed that overweight and obesity are common in adults with CP; however, BMI was not associated with vitamin D status. This is congruent with our previous work [20], and that of others [32] which demonstrates that indicators of central adiposity are potentially more sensitive for detecting standard clinical markers of cardiometabolic risk (e.g. triglycerides, HDL-cholesterol, total cholesterol to HDL cholesterol ratio, LDL-cholesterol, HOMA-IR, etc..) in adults with CP. Collectively, these findings support our ongoing contention that adults with CP are at higher risk for normal weight obesity and cardiometabolic dysregulation [16], and that indicators of central adiposity are superior to BMI for risk stratification. This is not surprising considering the mounting evidence in support of this diagnostic paradigm shift, even among the general population [33], and thus clinicians should be aware of the appropriate gender- and race/ethnicity-specific recommended waist circumference thresholds for abdominal obesity [34,35].

In the general population, recent data have also revealed a link between vitamin D and preservation of muscular strength [36]. This may have significant implications for individuals with CP over the lifespan, as even children with CP have impaired recruitment of target musculature during voluntary activity, and an over-recruitment and co-activation of antagonist musculature [37]. Premature sarcopenia and muscular weakness in CP are suggested to translate to acute functional deficit and disability [38]. As a result, functional loss, especially of mobility, is a major issue in adults with CP. The minority of individuals that actually report preservation of mobility throughout adulthood accredit this to maintenance of strength, balance and overall fitness. It is therefore critical to gain a greater understanding of how treatment of such modifiable risk factors as vitamin D deficiency could impact long term preservation of functional capacity and activity participation.

An obvious limitation in this study is that we could not account for dietary intake of calcium or vitamin D. Thus, it is possible that more severely affected individuals were adhering to a stricter regimen of nutrient supplementation (as is commonly recommended with anticonvulsant therapies), which would explain the lack of differences across GMFCS levels. Future research should account for nutrient intake, as well as any medication that may interfere with calcium and vitamin D. Further, as with all cross-sectional investigations, a limitation of this study is the inability to disentangle the cause-effect relationship between predictors and outcomes. Perhaps just as importantly, with this design we were unable to examine numerous potential mediators that were not measured. For example, no measures of muscle strength were made, and thus although vitamin D status was not associated with GMFCS in this study, it is possible that vitamin D status may affect muscle strength differently among individuals with more severe mobility impairments. It is also plausible that vitamin D deficiency may lead to diminished vitamin D receptor (VDR) in type II muscle fiber (see recent review [39]), and thus have a direct influence on muscular weakness, or conversely, strength improvement, with supplementation [40].

## Conclusions

This is one of the largest studies to examine vitamin D status in a heterogeneous sample of adults with CP, and to determine an independent, inverse association with abdominal obesity. Further, our finding that vitamin D status was not linked with GMFCS is novel and important, as degree of motor impairment tends to be the clinical scapegoat of secondary problems for this population. Unfortunately, there is still a large gap between the basic research intended to uncover novel etiologic factors and treatments of CP, and that which occurs at the translational level to understand secondary mechanisms or complications unique to this population. Clinical research tends to focus on common symptoms such as pain, spasticity, and mobility issues, and the viability and effectiveness of respective medical interventions. There has been very little attention directed at understanding the predictors of musculoskeletal pathology or cardiometabolic risk in patients with CP through the lifespan. Future research is certainly warranted to examine the appropriate timing and dosing of supplemental vitamin D in children and adults with CP, as well as identifying optimal behavioral interventions to reduce risk of abdominal obesity.

## Competing interests

The authors declare that they have no competing interests.

## Authors’ contributions

HH and EH carried out clinical testing and data collection. MP and AC drafted the manuscript. MP analyzed data and provided statistical interpretation. All authors read and approved the final manuscript.
